# Sequential butylphthalide therapy combined with dual antiplatelet therapy in the treatment of acute cerebral infarction

**DOI:** 10.12669/pjms.36.4.1831

**Published:** 2020

**Authors:** Xuewen Wo, Jinyan Han, Jiajia Wang, Xinmin Wang, Xiaoying Liu, Zhonggong Wang

**Affiliations:** 1 Xuewen Wo, Departments of Neurology, Binzhou People’s Hospital, Shandong, 256610, China; 2 Jinyan Han, Departments of Neurology, Binzhou People’s Hospital, Shandong, 256610, China; 3 Jiajia Wang, Departments of Neurology, Binzhou People’s Hospital, Shandong, 256610, China; 4 Xinmin Wang, Departments of Neurology, Binzhou People’s Hospital, Shandong, 256610, China; 5 Xiaoying Liu, Departments of Neurology, Binzhou People’s Hospital, Shandong, 256610, China; 6 Zhonggong Wang, Departments of Neurology, Binzhou People’s Hospital, Shandong, 256610, China

**Keywords:** Acute cerebral infarction, Butylphthalide, Dual antiplatelet, Sequential therapy

## Abstract

**Objective:**

To observe the clinical efficacy of sequential butylphthalide therapy combined with dual antiplatelet therapy in the treatment of elderly patients with acute cerebral infarction (ACI).

**Methods:**

One hundred and twenty-two elderly patients with ACI who were admitted to the department of neurology of our hospital at May 2016-August 2018 were selected grouped into a control group and an observation group by random number table method, 61 in each group. On the basis of conventional treatment, the patients in the control group were given dual antiplatelet therapy (aspirin enteric-coated tablets + clopidogrel bisulfate tablets), while the patients in the observation group were given sequential butylphthalide therapy on the basis of the control group. The clinical effects of the two groups were compared after four weeks of treatment, and the changes of National Institutes of Health Stroke Scale (NIHSS), ADL score, plasma 3-mercaptopyruvate sulphurtransferase (3-MST) and Amyloid β_42_ (Aβ_42_) levels and the occurrence of adverse reactions during treatment were recorded.

**Results:**

The clinical efficacy of the observation group was better than that of the control group (P<0.05). There was no significant difference in NIHSS and ADL scores between the two groups before treatment (P>0.05). After treatment, the NIHSS and ADL scores of the observation group were better than those of the control group (P<0.05). There was no significant difference in plasma levels of 3-MST and AB42 between the two groups before treatment (P>0.05). The level of plasma 3-MST in the observation group was higher than that in the control group, and the level of plasma Aβ42 was lower than that in the control group (P<0.05). No serious adverse reactions occurred during the treatment period in both groups.

**Conclusion:**

Butylphthalide sequential therapy combined with dual antiplatelet therapy is effective in the treatment of elderly ACI. It can effectively improve the plasma level of 3-MST and decrease the plasma level of Aβ42, which is conducive to improving the living ability and neurological function of patients and has high safety.

## INTRODUCTION

Acute cerebral infarction (ACI) is a common neurological disease. The incidence of ACI has been increasing in recent years.^1^ ACI, also known as ischemic stroke, is caused by blood supply disorders induced by various reasons in regional brain tissue, leading to necrosis of ischemic and hypoxic lesions in brain tissues and the clinical neurological deficits,^2^,^3^ ACI may cause limb function and language dysfunction and can also affect cognitive function of patients in varying degrees, manifested as mental and behavioral abnormalities, apraxia, agnosia, loss of understanding and so on, and it will cause serious harm to patients’ lives and living quality.^4^,^5^

ACI patients are often treated symptomatically by expanding blood volume, reducing intracranial pressure, anticoagulating and controlling basic diseases. However, in recent years, with the change of people’s lifestyle, the etiology of ACI is complex and diverse, and the effect of conventional treatment is poor.^6^,^7^ At present, thrombolysis and anti-platelet aggregation are commonly used in the treatment of ACI patients, and promoting the recovery of patients’ nervous function is one of the key points in the treatment of ACI.^8^,^9^ Aspirin combined with clopidogrel is the standard dual antiplatelet therapy scheme for ACI patients.^10^ Butylphthalide is the ACI treatment drug recommended by the Chinese Guidelines for the Diagnosis and Treatment of Acute Ischemic Stroke (2014).^11^ The latest research suggests that butylphthalide can significantly improve the neurological deficits of patients and improve their living ability.^12^

The purpose of this study was to observe the clinical efficacy of butylphthalide injection combined with dual antiplatelet therapy in elderly patients with ACI, in order to provide a reference for clinical effective treatment of ACI.

## METHODS

### General data

This is a retrospective study, and the size of sample was calculated using the following formula:


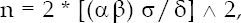


where δ stands for the required degree of distinction, σ stands for the standard deviation or its estimated value, s, α and β can refer to the row of degree of freedom v = ∞ in the table of t critical value.

One hundred and twenty-two elderly patients with ACI who were admitted to the Department of Neurology of our hospital at May 2016-August 2018 were selected according to the principle of randomization after the approval of Ethical Committee at October 15, 2019. All the patients met the diagnostic criteria of ACI in the Guidelines for Secondary Prevention of Ischemic Stroke and Transient Ischemic Attack of China (2014) and were diagnosed by imaging examination.^13^ All the patients were divided into a control group and an observation group by random number table method, 61 in each group. In the control group, there were 38 males and 23 females, with an average age of (65.31±7.56) years, and the average time between onset and admission was (24.51±4.09) h. As to complications, 30 cases had hypertension, 26 cases had diabetes and 33 cases had hyperlipidemia. In the observation group, there were 36 males and 25 females, with an average age of (63.63±6.56) years, and the average time between onset and admission was (25.72±4.81) h. As to complications, 35 cases had hypertension, 25 cases had diabetes, and 30 cases had hyperlipidemia. There were no significant differences in gender, age, time between onset and admission, and underlying diseases between the two groups (P>0.05); therefore, the results were comparable. The research program was approved by the Medical Ethics Committee of the hospital, and the patients or their families signed consent agreement.

### Inclusion criteria

Inclusive criteria included aging from 45 to 70 years old, cerebral infarction patients within onset within 6~24 h, and 5~25 points of National Institutes of Health Stroke Scale (NIHSS).

### Exclusion Criteria

1. Having serious basic diseases in the cardiac, pulmonary, liver and kidney, intracranial hemorrhage, malignant tumors or severe consciousness disorders.
2. Having coagulation dysfunction or bleeding tendency, or taking anticoagulant drugs.
3. Having allergic history.
4. Pregnant or lactating women.

### Therapeutic methods

Both groups were given conventional treatment after admission, including oral administration of 20 mg/d atorvastatin. Patients in the control group were given dual antiplatelet therapy on the basis of conventional treatment: orally taking aspirin enteric-coated tablets (Bayer SFDA approval number J20130078), 200 mg each time, once a day; orally taking clopidogrel bisulfate tablets (Sanofi (Hangzhou) SFDA approval number J20130083), 75 mg each time, once a day.

On the basis of the control group, patients in the observation group were intravenously injected with butylphthalide injection (Enbipu Pharmaceutical Co., Ltd., Shiyao Group, SFDA approval number H20100041), 100 mL/time, 2 times/day. Two weeks later, the patients were given butylphthalide capsule (Enbipu Pharmaceutical Co., Ltd., Shiyao Group, SFDA approval number H20050299), 0.2 g/time, 3 times/day. Both groups were treated for four weeks.

### Observation index

The clinical efficacies of two groups of patients were observed. Criteria for judging clinical efficacy included: marked effect: NIHSS score decreased by higher than 90% after treatment;^14^ effective: 18% ≤ decrease of NIHSS score < 90% after treatment; ineffective: NIHSS score decreased by lower than 18%; effective rate=(number of cases of marked effect+number of effective cases)/total number of cases×100%. The neurological function of patients was tested and evaluated using NIHSS. The lower the NIHSS score, the milder the neurological functional deficit. The ability of daily living was systematically evaluated using ADL scale (14 items). The higher the score, the more ideal the ability of daily living. 5 ml of fasting peripheral venous blood was taken from each patient before and after treatment, added with heparin sodium anticoagulation, and centrifuged at 3000 r/min for 10 minutes. The plasma was collected and stored at -80°C for testing. The levels of plasma 3-mercaptopyruvate sulphurtransferase (3-MST) and Amyloid β_42_ (Aβ_42_) were detected by enzyme-linked immunosorbent assay (ELISA) and Pulang DNM-9602G microplate reader. The kit was purchased from Shanghai Jianglai Biological Technology Co., Ltd. and operated strictly according to the kit instructions. The adverse reactions of the two groups during treatment were recorded.

### Statistical methods

SPSS 21.0 was used for data processing. The measurement data were expressed as (Mean±SD). The comparison between groups used two independent-sample t test. The counting data was expressed by relative number and processed by X^2^ test. The rank sum test was used for ranked data analysis. The difference was assessed as statistically significant if P<0.05.

## RESULTS

The effective rate of treatment in the observation group was 92.5%, which was significantly higher than that in the control group (78.0%). There was significant difference between the two groups (P<0.05, Table-I).Before treatment, there was no significant difference in NIHSS score and ADL score between the two groups (P>0.05). After treatment, the improvement of NIHSS and ADL scores in the observation group were significantly better than that in the control group, and the differences were statistically significant (P<0.05, Table-II).

**Table-I:** Clinical effects between two groups [n (%)].

Group	Markedly effective	Effective	Invalid	Effective rate
Observation group	44(72.13)	12(19.67)	5(8.20)	56(91.80)
Control group	34(55.74)	14(22.95)	13(21.31)	48(78.69)
X^2^	/	/	/	10.161
P	/	/	/	<0.05

**Table-II:** NIHSS score and ADL score between two groups before and after treatment.

Group	NIHSS score		ADL score	

	Before treatment	After treatment	Before treatment	After treatment
Observation group	12.7±5.9	5.9±3.7	15.6±6.3	52.3±14.8
Control group	12.8±3.4	8.6±4.1	15.6±5.2	32.6±12.2
t	0.423	6.931	0.374	8.312
P	>0.05	<0.05	>0.05	<0.05

There were no significant differences in plasma levels of 3-MST and Aβ_42_ between the two groups before treatment (P>0.05). After treatment, the plasma level of 3-MST in the observation group was higher than that in the control group, and the plasma level of Aβ_42_ was lower than that in the control group; the differences were statistically significant (P<0.05, Table-III).

**Table-III:** Plasma levels of 3-MST and Aβ42 between two groups before and after treatment.

Group	3-MST (μg/L)		A beta 42 (ng/L)	

	Before treatment	After treatment	Before treatment	After treatment
Observation group	1.70±0.23	4.45±0.18	59.72±5.14	30.66±3.63
Control group	1.65±0.21	3.06±0.35	60.40±5.26	42.51±4.83
t	1.217	7.671	0.703	12.917
P	>0.05	<0.05	>0.05	<0.05

During the intervention, there were four cases of significantly increased transaminase (< 80 U/L), two cases of abdominal discomfort, and one case of nausea in the control group, and the incidence of adverse reaction was 11.48%. In the observation group, there were four cases of significantly increased transaminase (< 80 U/L), two cases of abdominal discomfort and two cases of nausea, and the incidence of adverse reactions was 13.11%. There was no significant difference in the incidence of adverse reactions between the two groups (X^2^=0.29, P=0.59). All the above adverse reactions recovered normally after one week of drug withdrawal. All the patients had no adverse reactions such as gastrointestinal bleeding, subcutaneous hemorrhage, coagulation disorders and drug allergy.

## DISCUSSION

The onset of cerebral infarction is rather acute and without warning. After the occurence of cerebral infarction, serious blood circulation disorders will occur in brain tissue in a short time, leading to hypoxia and ischemia in brain tissue.^15^ Therefore, early recovery of cerebral blood supply and nerve function is the key to the treatment of ACI. Anticoagulation, thrombolysis and protection of nerve function are the main therapies. However, the best time window for thrombolysis in ACI patients is within 24 hours after onset. Most patients have missed the best time window for thrombolysis when they see a doctor; at that time, antiplatelet aggregation therapy is needed.^16^

Aspirin and clopidogrel are commonly used anti-platelet aggregation drugs in clinic, but their mechanisms of action are different. Therefore, the treatment of ACI with aspirin or clopidogrel alone has some limitations, while the effect of dual antiplatelet therapy is relatively definite.^17^,^18^ Butylphthalide is a new drug approved for market in 2004. It is a synthetic racemate, whose structure is similar to l-apigenin in celery seed. A relevant research shows that the use of butylphthalide in the treatment of stroke patients can block the inflammatory response of patients after cerebral ischemia and hypoxia from multiple channels and links and play an effective role in protecting and repairing patients’ brain neurons.^19^ The main functions of butylphthalide are: 1) to improve the ischemic tissue of brain tissue, promote blood flow, and reduce the degree of brain injury; 2) to effectively resist platelet aggregation and thrombosis, relieve brain edema and ischemic area, promote blood microcirculation of brain tissue, improve its energy metabolism, and achieve the effect of inhibiting neuronal apoptosis; 3) to effectively increase the content of nitric oxide (NO) and prostacyclin (PGI2) in cerebral blood vessels, reduce the content of arachidonic acid and the secretion of glutamate, alleviate the phenomenon of intracellular calcium overload, improve the activity of antioxidant enzymes, and reduce and inhibit the inflammatory response in the injured area.^20^,^21^ The sequential therapy of butylphthalide refers to choosing different treatment methods at proper time according to different conditions of patients, including using different drugs/different ways of medication. For ACI patients in critical condition, intravenous administration of butylphthalide in the early stage can help to alleviate the patient’s condition quickly; after two weeks of treatment, oral administration of butylphthalide cannot only maintain the effect of drug treatment, but also facilitate the treatment of patients at home after discharge. At present, butylphthalide has entered into phase IV clinical trials from phase I clinical trials, and its effectiveness and safety have been widely recognized.^22^-^24^

This study treated patients with progressive cerebral infarction using butylphthalide and sodium chloride injection in combination with dual anti-platelet therapy. The research results showed that the effective rate of treatment in the observation group was 92.5%, significantly higher than that in the control group (78.0%). There was significant difference between the two groups (P<0.05). NIHSS score in the observation group was significantly higher than that in the control group (P<0.05). The ADL score of the observation group was significantly lower than that of the control group, and the difference between the two groups was statistically significant (P<0.05). The above results showed that sequential treatment of butylphthalide could effectively promote the establishment of collateral circulation, improve the blood flow of ischemic penumbra, promote the recovery of nerve cells, improve the prognosis of patients and reduce the disability rate of cerebral infarction, which is similar to previous research results.^25^

A recent study has shown that hydrogen sulfide can improve neurons during cerebral ischemia and hypoxia,^26^ and it is beneficial to alleviate cerebral ischemia-reperfusion injury. 3-MST is the main enzyme producing hydrogen sulfide in the nervous system, and its plasma level is related to the degree of brain injury. Aβ_42_ is the main component of senile plaque, the brain ischemia and hypoxia can affect Aβ_42_, and its plasma level is closely related to the degree of nerve injury. The results of the present study showed that the plasma level of 3-MST in the observation group was higher than that in the control group after treatment, and the plasma level of Aβ_42_ was lower than that in the control group. It suggested that sequential butylphthalide therapy combined with dual antiplatelet therapy could effectively increase the content of hydrogen sulfide in elderly patients with ACI and reduce the content of Aβ_42_ to play a neuroprotective role and reduce neurotoxicity, which was consistent with Yang.^27^ In addition, no serious adverse reactions occurred during the treatment in both groups, suggesting that sequential butylphthalide therapy combined with dual antiplatelet therapy was safer for elderly ACI.

### Limitations of the Study

The sample size of this study was small, and moreover this study was single-center. The long-term prognosis was not further observed. Therefore, the number of cases should be increased in the future, and multi-center, long-term observation study is needed for further explication.

## CONCLUSION

Sequential butylphthalide therapy combined with dual antiplatelet therapy is effective in the treatment of elderly ACI. It can effectively improve the plasma level of 3-MST and decrease the plasma level of Aβ_42_. It is conducive to improving the neurological function and living ability of patients and has high safety; therefore, it is worthy of clinical application. However, the sample size of this study is small, and the long-term prognosis was not further observed. Therefore, we should increase the number of cases in future studies and look forward to further clarification of multi-center and long-term observation studies.

### Authors’ Contribution

**XWW, JYH & ZGW:** Study design, data collection and analysis.

**XWW, JJW, XMW & XYL:** Manuscript preparation, drafting and revising.

**XYL & ZGW:** Review and final approval of manuscript.

**XWW:** Responsible and accountable for the accuracy or integrity of the work
